# Supplementary material for: Effects of Osthol Isolated from Cnidium monnieri Fruit on Urate Transporter 1
Source: Molecules. 2018 Nov 1;23(11):2837. doi: 10.3390/molecules23112837 (PMC6278453; doi:10.3390/molecules23112837)
Supplement: Supplementary file 1 [file molecules-23-02837-s001.pdf]

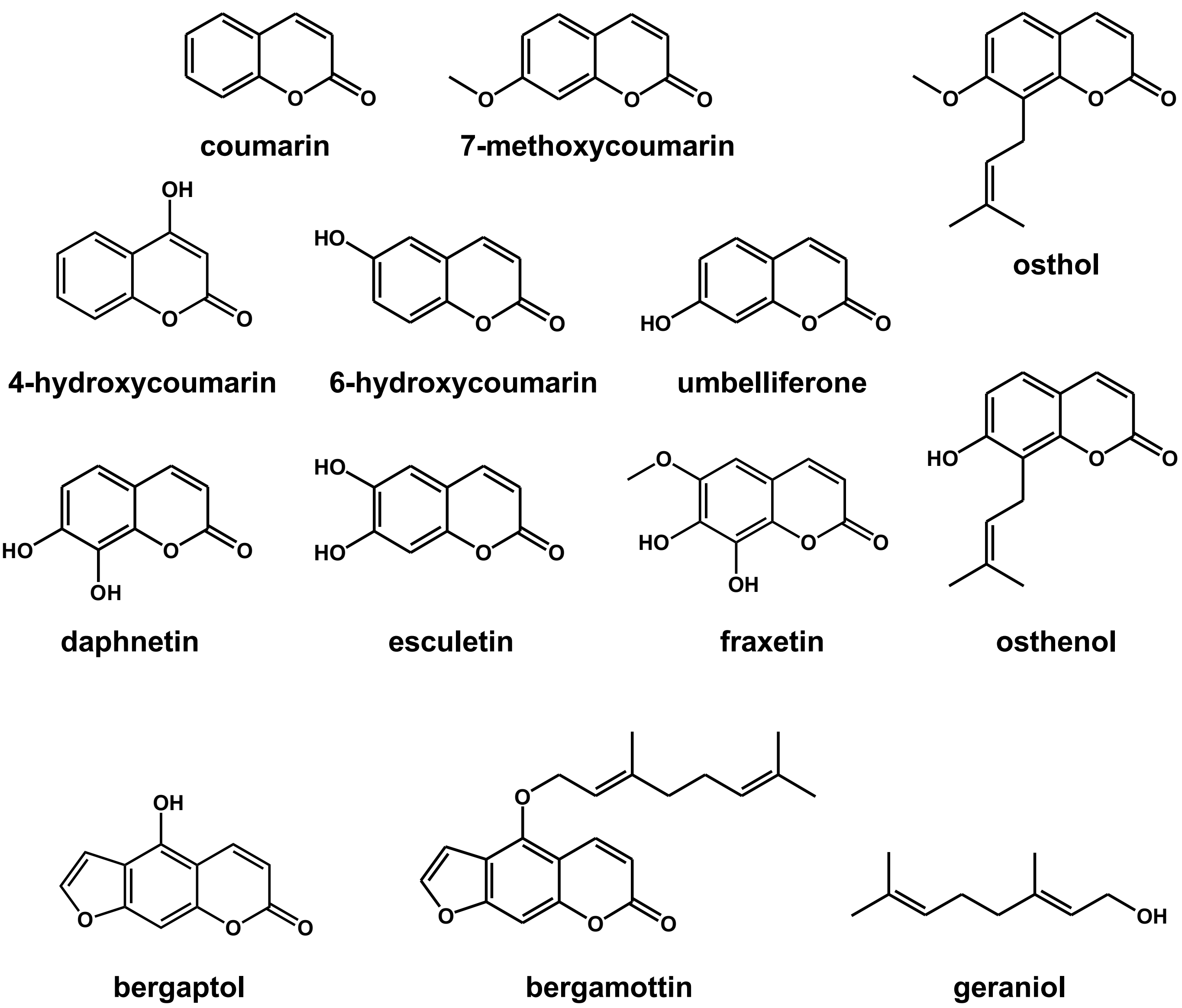

Figure S1. Chemical structures of the compounds used as samples in this study.

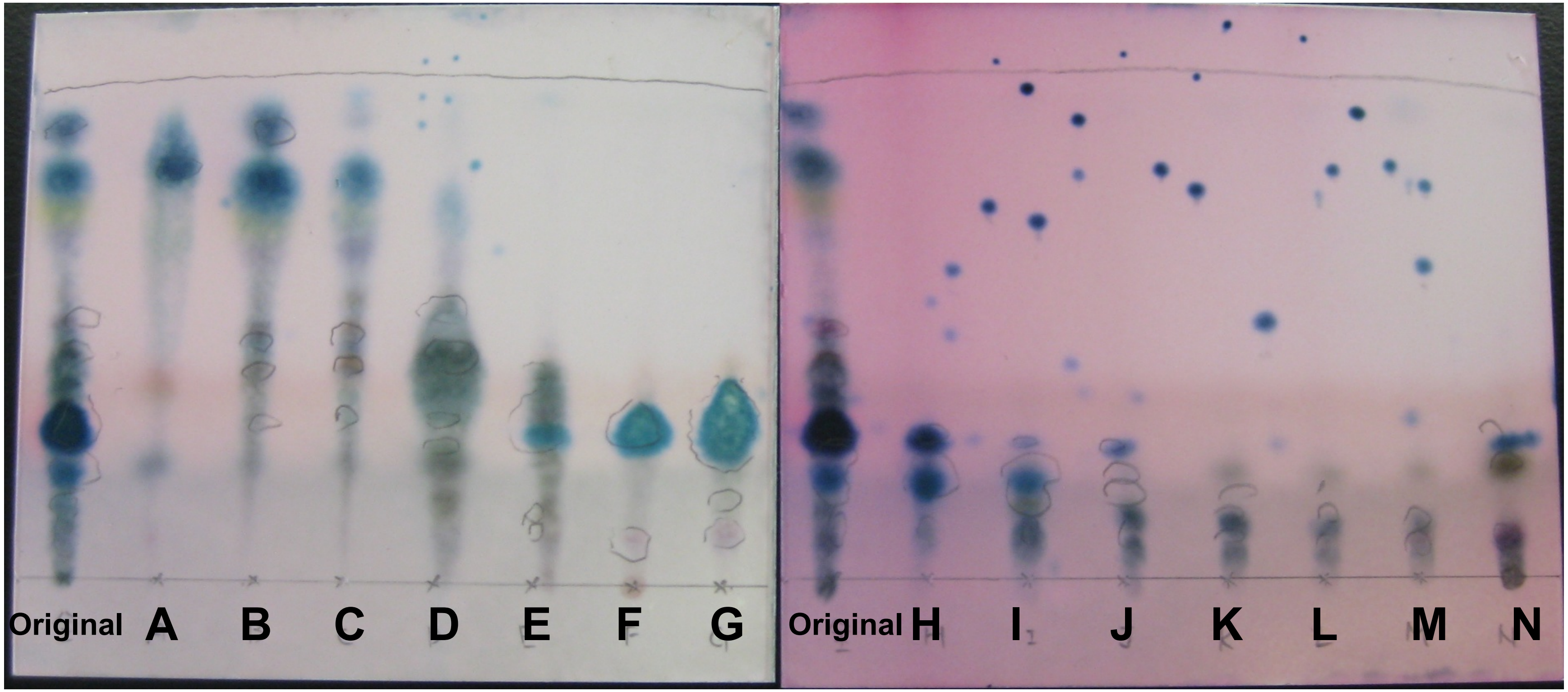

Figure S2. TLC pattern of the fractions collected from the hexane crude fraction. The hexane crude fraction (3.0 g) was applied to open silica gel chromatography eluted with hexane:acetone (8:2), and the fractions A–N were collected. Each fraction (3 µg) was spotted to TLC plate, spread out with hexane:acetone (8:2), and colored with anisaldehyde reagent. "Original" means the original hexane crude fraction. Large single spot in the fraction F is osthol.

Table S1. The origin, distributer name, lot number of the sample, and the ratio yielded of crude drugs used.

Latin name of crude drug	Origin	Distributer[a)]	Lot #	Ratio yielded (%)[b)]
Achyranthis Radix	The dried root of *Achyranthes bidentata* Blume	Tsumura	22026591	9.4
Akebiae Caulis	The dried climbing stem of *Akebia quinata* Decaisne	Tsumura	23006161	4.2
Alismatis Rhizoma	The dried tuber of *Alisma plantago-aquatica* subsp. *orientale* (Sampaio) Sampaio	Tsumura	22043631	6.8
Alpiniae Officinari Rhizoma	The dried rhizome of *Alpinia officinarum* Hance	Tsumura	23011641	9.0
Amomi Semen	The dried seed mass of *Amomum villosum* var. *xanthioides* (Wall. ex Baker) T.L.Wu & S.J.Chen	Tsumura	22042491	1.2
Anemarrhenae Rhizoma	The dried rhizome of *Anemarrhena asphodeloides* Bunge	Tsumura	23005341	16.8
Angelicae Dahuricae Radix	The dried root of *Angelica dahurica* Benth. et Hook. F. ex Franch. et Sav.	Tsumura	22041851	16.0
Angelicae Radix	The dried root of *Angelica acutiloba* (Siebold & Zucc.) Kitag.	Tsumura	22049651	11.5
Araliae Cordatae Rhizoma	The dried rhizome of *Aralia cordata* Thunberg	Tsumura	22006261	13.9
Arctii Fructus	The dried fruit of *Arctium lappa* Linné	Tsumura	21054451	2.9
Arecae Semen	The dried seed of *Areca catechu* Linné	Tsumura	22031171	2.8
Arisaematis Tuber	The dried tuber of *Arisaema heterophyllum* Blume	Tsumura	22038971	1.2
Armeniacae Semen	The dried seed of *Prunus armeniaca* Linné	Tsumura	22009451	7.7
Artemisiae Capillaris Flos	The dried capitulum of *Artemisia capillaris* Thunberg	Tsumura	22036091	8.6
Artemisiae Folium	The dried leaf and twig of *Artemisia princeps* Pampanini	Tsumura	22031681	10.1
Asiasari Radix	The dried root with rhizome of *Asiasarum sieboldii* F. Maekawa	Tsumura	22020521	7.7
Asparagi Radix	The dried root of *Asparagus cochinchinensis* Merrill	Tsumura	23011471	14.1
Astragali Radix	The dried root of *Astragalus propinquus* Schischkin	Tsumura	22043331	17.3
Atractylodis Lanceae Rhizoma	The dried rhizome of *Atractylodes lancea* De Candolle	Tsumura	23001771	16.6
Atractylodis Rhizoma	The dried rhizome of *Atractylodes japonica* Koidzumi ex Kitamura	Tsumura	23018581	23.8
Aurantii Fructus Immaturus	The dried immature fruit of *Citrus* x *aurantium* Linné	Tsumura	22043361	13.3
Aurantii Nobilis Pericarpium	The dried ripe fruit skin of *Citrus reticulata* Blanco	Tsumura	23002241	35.3
Bambusae Caulis	The dried culm of *Bambusa textilis* McClure	Tsumura	22036071	3.7
Benincasae Semen	The dried seed of *Benincasa hispida* (Thunberg) Cogniaux	Tsumura	21057771	8.2
Bupleuri Radix	The dried root of *Bupleurum falcatum* Linné	Tsumura	22025091	8.8

Cannabis Fructus	The dried fruit of *Cannabis sativa* Linné	Tsumura	23018661	2.4
Carthami Flos	The dried tubulous flower of *Carthamus tinctorius* Linné	Tsumura	22040711	14.2
Caryophylli Flos	The dried flowering bud of *Syzygium aromaticum* (Linné) Merrill et Perry	Tsumura	22000321	14.5
Chrysanthemi Flos	The dried capitulum of *Chrysanmum morifolium* Ramatulle	Tsumura	22008841	10.6
Cicadae Periostracum	The dried cicada shell of *Cryptotympana atrata* Stal	Daiko	5C30	1.0
Cimicifugae Rhizoma	The dried rhizome of *Actaea simplex* (DC.) Wormsk. ex Prantl	Tsumura	21017241	5.1
Cinnamomi Cortex	The dried bark of *Cinnamomum cassia* (Linné) J.Presl	Tsumura	22044881	2.6
Clematidis Radix	The dried dried root of *Clematis chinensis* Osbeck	Tsumura	21020771	7.5
Cnidii Monnieris Fructus	The dried fruits of *Cnidium monnieri* (Linné) Cusson	Tochimoto	23610001	2.9
Cnidii Rhizoma	The dried rhizome of *Cnidium officinale* Makino	Tsumura	23001371	9.7
Coicis Semen	The dried seed of *Coix lachryma-jobi* var. ma-yuen (Rom.Caill.) Stapf, from which the seed coat has been removed	Tsumura	23018091	4.0
Coptidis Rhizoma	The dried rhizome of *Coptis japonica* (Thunberg) Makino	Tsumura	22048301	8.5
Corni Fructus	The dried pseudocarp of *Cornus officinalis* Siebold et Zuccarini	Tsumura	22038491	37.3
Corydalis Tuber	The dried tuber of *Corydalis turtschaninovii* Basser	Tsumura	22042871	0.4
Cyperi Rhizoma	The dried rhizome of *Cyperus rotundus* Linné	Tsumura	22005381	3.2
Dioscoreae Rhizoma	The dried rhizome of *Dioscorea japonica* Thunberg	Tsumura	23000221	2.0
Ephedrae Herba	The dried terrestrial stem of *Ephedra sinica* Stapf	Tsumura	22050411	11.2
Eriobotryae Folium	The dried leaf of *Eriobotrya japonica* (Thunberg) Lindley	Tsumura	23010081	13.7
Euodiae Fructus	The dried fruit of *Tetradium ruticarpum* (A.Juss.) T.G.Hartley	Tsumura	22038071	7.2
Foeniculi Fructus	The dried fruit of *Foeniculum vulgare* Miller	Tsumura	21023321	2.0
Forsythiae Fructus	The dried fruit of *Forsythia suspensa* (Thunberg) Vahl	Tsumura	23012601	6.9
Fossillia Ossis Mastodi	The ossified bone of large mammal, and is mainly composed of calcium carbonate	Tsumura	23006231	0.6
Fritillariae Bulbus	The dried bulb of *Fritillaria thunbergii* Miquel	Tsumura	23012111	1.1
Fructus Hordei Garminatus	The dried ripe cariopsis of *Hordeum vulgare* Linné	Daiko	7K27	5.4
Gardeniae Fructus	The dried fruit of *Gardenia jasminoides* J. Ellis	Tsumura	22031161	18.2
Gastrodiae Tuber	The dried steamed tuber of *Gastrodia elata* Blume	Tsumura	23011631	1.4
Gentianae Scabrae Radix	The root and rhizome of *Gentiana scabra* Bunge	Tsumura	22009991	28.8
Geranii Herba	The dried terrestrial part of *Geranium thunbergii* Siebold ex Lindl. & Paxton	Daiko	Not shown	5.2

Ginseng Radix	The dried root of *Panax ginseng* C.A.Mayer	Tsumura	23018601	7.8
Glycyrrhizae Radix	The dried root and stolon of *Glycyrrhiza uralensis* Fisher	Tsumura	22053201	18.8
Houttuyniae Herba	The dried the terrestrial part of *Houttuynia cordata* Thunberg	Daiko	Not shown	3.2
Lilii Bulbus	The dried scaly leaves of *Lilium lancifolium* Thunberg	Tsumura	22029511	0.2
Linderae Radix	The dried root of *Lindera strychnifolia* Fernandez-Villar	Daiko	8C25	3.3
Longan Arillus	The dried aril of *Euphoria longana* Lamarck	Tsumura	22036971	31.2
Lonicerae Folium Cum Caulis	The dried leaves and stems of *Lonicera japonica* Thunberg	Tsumura	22040701	6.6
Lycii Cortex	The dried root skin of *Lycium barbarum* Linné	Tsumura	23000421	3.1
Magnoliae Cortex	The dried bark of *Magnolia obovata* Thunberg	Tsumura	23012431	6.9
Magnoliae Flos	The dried flower bud of *Magnolia salicifolia* Maximowicz	Tsumura	22028611	13.9
Menthae Herba	The dried terrestrial part of *Mentha canadensis* Linné	Tsumura	22044091	10.6
Mori Cortex	The dried root bark of *Morus alba* Linné	Tsumura	22034951	5.8
Moutan Cortex	The dried root bark of *Paeonia suffruticosa* Andrews	Tsumura	23005351	11.0
Nelumbis Semen	The dried seed of *Nelumbo nucifera* Gaertner	Tsumura	23006011	4.6
Notopterygii Rhizoma	The dried rhizome and root of *Notopterygium incisum* Ting ex H. T. Chang	Tsumura	22041601	15.5
Nupharis rhizoma	The dried longitudinally split rhizome of *Nuphar japonicum* De Candolle	Tsumura	20009891	6.2
Ophiopogonis Radix	The dried root of *Ophiopogon japonicus* (Thunberg) Ker Gawler	Tsumura	23003151	8.7
Oryzae Fructus	The dried caryopsis of *Oryza sativa* Linné	Daiko	5G29	0.9
Ostreae Testa	The dried the shell of *Ostrea gigas* Thunberg	Tsumura	23014111	0.4
Paeoniae Radix	The dried root of *Paeonia lactiflora* Pallas	Tsumura	22011231	14.4
Paeoniae Radix Rubra	The dried root of *Paeonia anomala* subsp. *veitchii* (Lynch) D.Y.Hong & K.Y.Pan	Tsumura	22040921	7.5
Panacis Japonici Rhizoma	The dried root of *Panax japonicus* (T.Nees) C.A. Mayer	Tsumura	21018391	8.0
Perillae Herba	The dried tips of branches of *Perilla frutescens* var. *crispa* (Thunberg) H. Deane	Daiko	8F16	9.3
Persicae Semen	The dried seed of *Prunus persica* (Linné) Batsch	Tsumura	22052901	13.4
Phellodendri Cortex	The dried bark of *Phellodendron amurense* Ruprecht	Tsumura	22038081	11.5
Pinelliae Tuber	The dried tuber of *Pinellia ternata* (Thunberg) Makino	Tsumura	22042141	1.1
Plantaginis Semen	The dried seed of *Plantago asiatica* Linné	Tsumura	22049181	0.7
Platycodi Radix	The dried root of *Platycodon grandiflorum* (Jacques) A.De Candolle	Tsumura	22040381	7.7

Polygalae Radix	The dried root bark of *Polygala tenuifolia* Willdenow	Tsumura	22000701	16.6
Polygoni Multiflori Radix	The dried root of *Polygonum multiflorum* Thunberg	Tsumura	22039451	5.9
Polyporus	The dried sclerotium of *Polyporus umbellatus* Fries	Tsumura	22045121	1.0
Poria	The dried sclerotium of *Wolfiporia cocos* Ryvarden et Gilbertson	Tsumura	23007381	1.1
Processi Aconiti Radix	The dried tuberous root of *Aconitum carmichaeli* Debeaux prepared by autoclaving	Uchida	8511606	5.8
Processi Rehmanniae Radix	The dried root of *Rehmannia glutinosa* (Gaertner) A.De Candolle with the application of steaming	Tsumura	22054311	28.9
Puerariae Radix	The dried root of *Pueraria montana* var. *lobata* (Willd.) Sanjappa & Pradeep	Tsumura	22040931	10.8
Quercus Cortex	The dried bark of *Quercus acutissima* Carruthers	Tsumura	23001531	5.9
Rehmanniae Radix	The dried root of *Rehmannia glutinosa* (Gaertner) A.De Candolle	Tsumura	22029541	19.9
Rhei Rhizoma	The dried rhizome of *Rheum palmatum* Linné	Tsumura	22041591	26.0
Saposhnikoviae Radix	The dried root and rhizome of *Saposhnikovia divaricata* Schischkin	Tsumura	22049031	18.9
Saussureae Radix	The dried root of *Aucklandia lappa* de Candolle	Tsumura	23004371	11.7
Schisandrae Fructus	The dried fruit of *Schisandra chinensis* Baillon	Tsumura	22026581	25.0
Schizonepetae Spica	The dried spike of *Nepeta tenuifolia* Bentham	Tsumura	22042881	4.8
Scutellariae Radix	The dried root of *Scutellaria baicalensis* Georgi	Tsumura	22037381	10.4
Sinomeni Caulis et Rhizoma	The dried climbing stem and rhizome of *Sinomenium acutum* Rehder et Wilson	Tsumura	23008241	9.4
Sophorae Radix	The dried root of *Sophora flavescens* Aiton	Tsumura	22011021	9.2
Tribuli Fructus	The dried fruit of *Tribulus terrestris* Linné	Tsumura	22028771	2.2
Trichosanthis Radix	The dried root of *Trichosanthes kirilowii* Maximowicz	Tsumura	22029491	0.7
Trichosanthis semen	The dried seed of *Trichosanthes kirilowii* Maximowicz	Tsumura	22037861	9.1
Uncariae Uncis Cum Ramulus	The dried hook-bearing stem of *Uncaria rhynchophylla* Miquel ex Haviland	Tsumura	22045681	5.3
Zanthoxyli Piperiti Pericarpium	The dried ripe fruit of *Zanthoxylum piperitum* De Candolle	Tsumura	22004261	10.7
Zingiberis Rhizoma	The dried rhizome of *Zingiber officinale* Roscoe	Tsumura	23002281	4.2
Zingiberis Rhizoma Processum	The dried rhizome of *Zingiber officinale* Roscoe, after being steamed	Tsumura	22046221	7.3
Zizyphi Fructus	The dried fruit of *Ziziphus jujuba* Miller	Tsumura	22047121	60.3
Zizyphi Spinosi Semen	The dried seed of *Ziziphus jujuba* var. *spinosa* (Bunge) Hu ex H.F. Chou	Tsumura	23006931	4.5

Latin names of crude drugs were according to Japanese Pharmacopoeia 17th Edition (Pharmaceutical and Medical Device Regulatory Science Society of Japan, 2016) and non-Pharmacopoeial crude drugs 2015 (Pharmaceutical and Medical Device Regulatory Science Society of Japan, 2015). All crude drug samples met the grade standards of these books.

The quality managers of each distributer identified and certificated the plant species, and the voucher specimens are deposited in Department of Pharmacognosy, Graduate School of Pharmaceutical Sciences, Nagoya City University, Nagoya, Japan.

[a)]Daiko is Daiko Shoyaku (Nagoya, Japan). Tsumura is Tsumura & Co. (Tokyo, Japan). Tochimoto is Tochimoto Tenkaido Co. Ltd. (Osaka, Japan). Uchida is Uchida Wakanyaku Co., Ltd. (Tokyo, Japan)

[b)]Ratio yielded (%) is the ratio of the weight of MeOH extract to the weight of original crude drug.
